# No evidence for amyloid pathology as a key mediator of neurodegeneration post-stroke - a seven-year follow-up study

**DOI:** 10.1186/s12883-020-01753-w

**Published:** 2020-05-08

**Authors:** Guri Hagberg, Hege Ihle-Hansen, Brynjar Fure, Bente Thommessen, Håkon Ihle-Hansen, Anne Rita Øksengård, Mona K. Beyer, Torgeir B. Wyller, Ebba Gløersen Müller, Sarah T. Pendlebury, Per Selnes

**Affiliations:** 1 Bærum Hospital, Vestre Viken Hospital Trust, N-3004 Drammen, Norway; 2 Institute of Clinical Medicine, University of Oslo, Oslo, Norway; 3 Department of Geriatric Medicine, Oslo University Hospital, Oslo, Norway; 4 Department of Neurology, Department of Internal Medicine, Central Hospital Karlstad and Faculty of Medicine, Örebro University, Örebro, Sweden; 5 Department of Neurology, Akershus University Hospital, Oslo, Norway; 6 Division of Radiology, Nuclear Medicine Oslo University Hospital, Oslo, Norway; 7 Department of Nuclear Medicine, Oslo University Hospital, Oslo, Norway; 8 Centre for Prevention of Stroke and Dementia, Nuffield Department of Clinical Neurosciences, University of Oxford, Oxford, UK; 9 NIHR Oxford Biomedical Research Centre, John Radcliffe Hospital, Oxford, UK

**Keywords:** Stroke, Cognitive impairment, Cerebrospinal fluid, Positron emission tomography, Prognosis

## Abstract

**Background:**

Cognitive impairment (CI) with mixed vascular and neurodegenerative pathologies after stroke is common. The role of amyloid pathology in post-stroke CI is unclear. We hypothesize that amyloid deposition, measured with Flutemetamol (^18^F-Flut) positron emission tomography (PET), is common in seven-year stroke survivors diagnosed with CI and, further, that quantitatively assessed ^18^F-Flut-PET uptake after 7 years correlates with amyloid-β peptide (Aβ_42_) levels in cerebrospinal fluid (CSF) at 1 year, and with measures of neurodegeneration and cognition at 7 years post-stroke.

**Methods:**

208 patients with first-ever stroke or transient Ischemic Attack (TIA) without pre-existing CI were included during 2007 and 2008. At one- and seven-years post-stroke, cognitive status was assessed, and categorized into dementia, mild cognitive impairment or normal. Etiologic sub-classification was based on magnetic resonance imaging (MRI) findings, CSF biomarkers and clinical cognitive profile. At 7 years, patients were offered ^18^F-Flut-PET, and amyloid-positivity was assessed visually and semi-quantitatively. The associations between ^18^F-Flut-PET standardized uptake value ratios (SUVr) and measures of neurodegeneration (medial temporal lobe atrophy (MTLA), global cortical atrophy (GCA)) and cognition (Mini-Mental State Exam (MMSE), Trail-making test A (TMT-A)) and CSF Aβ_42_ levels were assessed using linear regression.

**Results:**

In total, 111 patients completed 7-year follow-up, and 26 patients agreed to PET imaging, of whom 13 had CSF biomarkers from 1 year. Thirteen out of 26 patients were diagnosed with CI 7 years post-stroke, but only one had visually assessed amyloid positivity. CSF Aβ_42_ levels at 1 year, MTA grade, GCA scale, MMSE score or TMT-A at 7 years did not correlate with ^18^F-Flut-PET SUVr in this cohort.

**Conclusions:**

Amyloid binding was not common in 7-year stroke survivors diagnosed with CI. Quantitatively assessed, cortical amyloid deposition did not correlate with other measures related to neurodegeneration or cognition. Therefore, amyloid pathology may not be a key mediator of neurodegeneration 7 years post-stroke.

**Trial registration:**

Clinicaltrials.gov (NCT00506818). July 23, 2007. Inclusion from February 2007, randomization and intervention from May 2007 and trial registration in July 2007.

## Background

Post-stroke cognitive impairment (CI) is caused by both vascular and neurodegenerative changes [1], and as Alzheimer disease (AD) accounts for 50–70% of all dementia cases [2], the role of AD pathology are important to address in CI post-stroke. Due to an aging population and a decline in post-stroke mortality, strategies for timely diagnosis and disease prevention are urgently needed [3–6]. Post-stroke cognitive impairment follows different trajectories, and risk scores based on clinical and neuroimaging variables are promising [7, 8], but do not include amyloid biomarkers. Amyloid positivity has been associated with more severe cognitive decline post-stroke in small studies [9, 10], and might be included in future prediction models and personalized management.

Known risk factors for dementia in the general population include neuroimaging variables such as β-amyloid deposition, medial temporal lobe atrophy (MTLA), and small vessel disease. The risk factors are additive, and there are several indications of mechanistic interactions [11, 12]. Recent studies support the use of amyloid positron emission tomography (PET) in patients with uncertain diagnosis in a memory clinic setting [13–15], but such studies are missing in the post-stroke population. Biomarkers of cortical amyloid deposition include cerebrospinal fluid (CSF) Amyloid-β peptide (Aβ_42_) levels and amyloid-binding PET tracers. CSF Aβ_42_ levels are established biomarkers of Alzheimer’s disease, and constitute the central biomarker for amyloid plaque formation [16]. ^18^F-Flutemetamol (^18^F-Flut) is increasingly used in dementia diagnostics, as it exhibits high affinity binding for fibrillary amyloid [17, 18]. A negative ^18^F-Flut-PET examination indicates sparse or no fibrillar amyloid, incompatible with a neuropathological diagnosis of AD [19]. ^18^F-Flut-PET and CSF Aβ_42_ levels are inversely highly correlated [15, 20–22]. In animal models, there has been a synergistic relationship between inflammation induced by stroke-related ischemia and β-amyloid deposits [23–25], but this association has not been studied in humans [26, 27]. Amyloid deposition increases with age [12], and theoretically might accelerate due to inflammation related to stroke. Quantitative assessment of amyloid PET allows a continuous approximation of amyloid plaques in different cortical regions and might be more informative than binary visual assessments. MTLA and cortical atrophy are well described in both AD and pure cerebrovascular disease [28–30], but there is still no consensus regarding the contribution of amyloid pathology to post-stroke neurodegeneration and cognitive impairments [31–33]. A recent study from Pendlebury et al. shows that Apolipoprotein E ε4 genotype, a known risk factor for AD linked to failure in amyloid clarence [34], is associated with increased risk of dementia after stroke [35]. The Atherosclerosis Risk in Communities (ARIC)-PET Amyloid Imaging Study showed that an increasing number of midlife vascular risk factors were associated with amyloid deposition, consistent with a role of vascular disease in the development of AD [36].

The reported prevalence of visually assessed amyloid positivity rage from 5% of (a total of 38) MCI patients assessed 6 months post-stroke [37], to 40% of (a total of 10) post-stoke dementia patients assessed from 0.5 to 4 years post-stroke [38]. Of the seven studies identified [9, 10, 32, 33, 38, 39], six used the amyloid tracer Pittsburg compound B, one used ^18^F-Flut-PET [37].

In the CAST (Cognition After STroke) study, dementia and mild cognitive impairment (MCI) were diagnosed and sub-classified according to proposed underlying etiology at one- and seven- years post-stroke [40, 41]. In the present sub-study from the CAST cohort, all stroke survivors were offered ^18^F-Flut-PET at seven-year follow-up. To our knowledge, this is the first study on ^18^F-Flut-PET 7 years post-stroke.

As stroke and AD share a common set of vascular risk factors, and amyloid depositions increases over time [42], we hypothesized that amyloid deposition is common in stroke survivors diagnosed with CI and, further, that quantitatively assessed ^18^F-Flut-PET uptake at 7 years post-stroke correlates with CSF Aβ_42_ levels at 1 year, and with measures of neurodegeneration and cognition at 7 years.

## Methods

### Participants

All patients with a first-ever TIA or stroke, without known cognitive decline pre-stroke, admitted to the stroke unit at Bærum Hospital between February 2007 and July 2009 were invited to participate in the CAST study. Patients with previous stroke or TIA, subarachnoid hemorrhage, life expectancy of less than 1 year, known cognitive decline as indicated by a score of ≥3.44 on The Informant Questionnaire on Cognitive Decline in the Elderly (IQCODE) [43], or patients who did not speak Norwegian were excluded. Stroke survivors were invited to participate in a follow-up study from February 2014 to July 2016. Details can be found in previous published papers [40, 44].

### Examinations and assessments

At baseline, stroke etiology was classified according to the Trial of ORG 10172 (TOAST) classification [45], and patients underwent neuroimaging with computed tomography (CT) and/or magnetic resonance imaging (MRI).

In short, at baseline, at one and at seven-year post-stroke, vascular risk factors, and current medication were recorded. Fasted blood samples and an electrocardiography (ECG), were obtained, and body mass index (BMI) was calculated. Neurological impairment was assessed using the National Institutes of Health Stroke Scale (NIHSS) [46]. Cognitive evaluation included the Mini Mental State Examination (MMSE) [47], the clock drawing test [48], the Trail making test part A and B (TMT-A and B) [49], and the 10-word memory test [50]. Additional tests at 7 year post-stroke were the Montreal Cognitive Assessment (MoCA) [51] and Controlled Oral Word Association Test (COWAT) [52]. Global functional outcome was assessed by the modified Rankin Scale (mRS) [53] and personal activities of daily living (p-ADL) by the Barthel Activities of Daily Living Index [53].

Supplementary investigations at one and 7 years included MRI of the brain, carotid ultrasound, and when possible, lumbar puncture was performed. Cerebrospinal fluid (CSF) was collected in polypropylene tubes and immediately transported to the local laboratory, in accordance with the manufacturers’ instructions. Biomarkers for neurodegenerative disease (amyloid-β peptide (Aβ_42_) levels, total tau (T-tau) and phosphorylated tau (P-tau)) were quantified with commercially available ELISAs (Fujirebio Europe, Gent, Belgium). The laboratory recommended a cut-off value of Aβ_42_ ≤ 550 ng/L for abnormality, modified from Sjögren et al. [54].

### Cerebral MRI

At one- and seven- years follow-up, MRI scans were acquired on a Philips Intera system 1.5 T (Philips Medical Systems, Best, The Netherlands). The MRI study protocol consisted of 3D-T1, axial T2, 3D-FLAIR, DWI and SWI sequences.

MRI investigations were evaluated for focal vascular lesions, medial temporal lobe atrophy (MTLA), white matter lesions (WML), and global cortical atrophy (GCA), by two radiologists, blinded to the clinical information. Any discrepancies were resolved by consensus. MTLA was graded from 0 to 4; with MTLA grade 0 = no atrophy, MTLA 4 = highest degree of atrophy. MTLA 0–1 is considered normal [55]. WML was rated using the visual rating scale proposed by Fazekas, scores ranging from 0 to 3 [56]. GCA was rated using the visual rating scale known as Pasquier scale, ranging from 0 to 3 [57].

### MRI segmentations and analyses

﻿Cortical reconstruction and volumetric segmentation were performed with the FreeSurfer image analysis suite version 6.0.0 (http://surfer.nmr.mgh. harvard.edu/). This includes segmentation of the subcortical white-matter and deep gray-matter volumetric structures [58] and parcellation of the cortical surface [59]. This labels cortical sulci and gyri, and thickness values are calculated in the regions of interest. All segmentations were manually inspected.

### ^18^F-Flutemetamol PET CT acquisition

Seven years post-stroke, patients were examined with a Siemens Biograph40 mCT scanner (Siemens Healthineers, Erlangen, Germany). All patients received an intravenous injection of approximately 185 MBq ^18^F-Flutemetamol (mean 188 MBq, range 165–218 MBq). Image acquisition started approximately 90 min after injection (mean 91 min, range 78–108) with a low dose CT followed by PET acquisition for 20 min (four frames of 5 minutes). 3D dynamic emission data were obtained with a resolution recovery algorithm with time of flight (TrueX with two iterations, 21 subsets and a Gaussian filter with FWHM of 2 mm, matrix size 400 × 400). Reconstructed images had a slice thickness of 2 mm and a voxel size of 2 × 2 × 2 mm^3^.

### Qualitative classification of ^18^F-Flutemetamol PET

^18^F-Flut PET images were visually classified as positive (^18^F-Flut-PET (+)) or negative (^18^F-Flut-PET (−)) by at least two nuclear medicine physicians with experience in ^18^F-Flut PET, and recorded in patients’ medical records in line with standard clinical practice between 2015 and 2017. In addition, one nuclear medicine physician with experience in ^18^F-Flut PET repeated classification in 2019, and in case of discrepancy between the classifications, an additional expert was consulted. All image classifications were performed according to the validated electronic reader program [60] and as described previously [15].

### Quantitative classification of ^18^F-Flutemetamol PET

Motion correction of the ^18^F-Flut PET was performed using frame-by-frame rigid registration, then the frames were summed to a single time-frame image and rigidly registered to the anatomical MRI volume using a 6-parameter rigid registration as implemented in the Statistical Parametrical Mapping (SPM 12, Wellcome Trust Centre for Neuroimaging, UCL, UK) toolbox. ^18^F-Flut-PET standardized uptake value ratios (SUVr) were obtained by normalization to the brainstem. Both the cerebellar cortex, pons and brainstem are widely used Flutemetamol reference regions, acquiring amyloid build plaques not until the fifth and final phase of amyloid deposition [61]. Our choice of reference region was influenced by local tradition, a feeling that the structural masks of the cerebellar cortex were less accurate, and a long series of publications utilizing the brainstem or pons as reference region. Prior to normalization, the brainstem mask was eroded by 1 mm to avoid partial volume effects, inaccurate segmentation or co-registration. ^18^F-Flut-PET uptake was analyzed in five pre-selected cortical regions of interest (ROIs), as defined by Desikan et al. [62] and implemented in FreeSurfer as described above, known to hold substantial amyloid plaques in AD: the precuneus and posterior cingulate combined, anterior cingulate, prefrontal, inferior parietal, and lateral temporal cortex [63]. The ^18^F-Flut-PET uptake for each region was averaged across the hemispheres. We further calculated a composite SUVr by averaging the uptake in the above-mentioned regions. SUVr were not calculated to categorize scans as positive or negative but used as continuous variables in correlation analyses.

### Outcomes and diagnosis of cognitive function

﻿The criteria outlined by Winblad et al. [64] were used for MCI, and the International Classification of Diseases 10th revision (ICD-10) criteria [2] for dementia diagnoses at both one and 7 years post-stroke. The diagnoses were made in consensus meetings by two senior neurologists (B.F and B.T) and one senior geriatrician (A.R.Ø). Details of the novel method for sub-classification have previously been reported [41], with six potential subgroups; degenerative MCI or degenerative dementia, vascular MCI (MCI VaD) or vascular dementia (dementia VaD) or mixed degenerative and vascular MCI or dementia. The evaluations were based on the results from the medical history, cognitive assessments, the IQCODE and information regarding daily functioning. Sub-classification for proposed underlying etiology was based on MRI findings of vascular or degenerative brain changes, biomarkers in the CSF, the patient’s vascular risk factors and clinical cognitive profile. Patients were classified with possible vascular disease when the radiological findings revealed WMLs without MTLA, while MTLA without WMLs was interpreted as degenerative etiology. Patients with combined pathologies were classified with mixed vascular and degenerative disease.

### Statistics

Categorical variables were compared with Pearson’s chi-squared test and continuous variables with independent Student t-test. The relationship between death as dependent variable and age-adjusted CSF as independent variables were assessed with logistic regression analyses. Correlation analyses of ^18^F-Flut-PET SUVr and measures of neurodegeneration and cognition, were performed assessing the Pearson correlation coefficient in continuous variables (MTA, CSF Aβ_42_ levels, GCA, TMT-A), Spearman rho in ranked data (MMSE). Linear regression analyses were performed with ^18^F-Flut-PET SUVr as explanatory variable. Each regression model was adjusted for age. Statistical analyses were performed using SPSS Statistic version 23.

## Results

### Study population

**Fig. 1:**
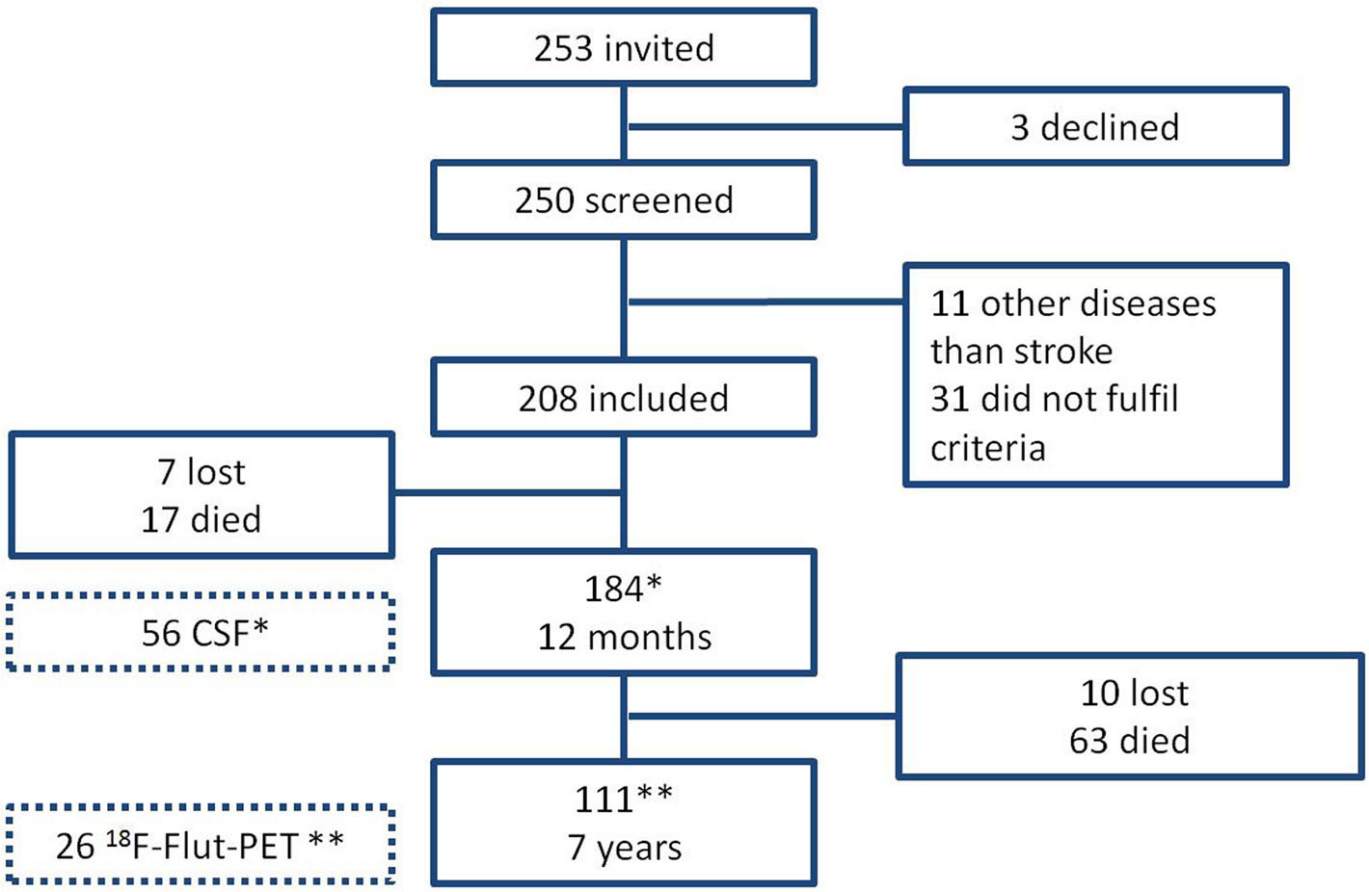
Flow chart

Of the 208 patients included in 2007–2008, 111 completed 7 years follow-up, of whom 26 patients agreed to PET imaging and 13 of these had CSF from 12 months. 12 out of the 26 patients who agreed to PET imaging were diagnosed with CI at 7 years. In total, 80 patients died during the study period, and 17 were lost to follow-up for other reasons. A flow chart is presented in Fig. 1. Characteristics and assessments at baseline and 7 years for the complete study population are presented in Table 1. Characteristics and assessment at baseline, clinical cognitive profile and MRI findings at 1 year in stroke survivors by PET or no PET at 7 years are presented in Table 2. Significantly more patients who agreed to PET had normal cognition and lower CSF T-tau at 12 months follow-up. There were no differences regarding age, stroke subtype or assessments at baseline, or MRI findings at 1 year.

**Table 1:** Baseline characteristics (*n* = 208) and assessments in survivors at 7 years (*n* = 111)

	Baseline	7-year follow-up
Male (%) *Mean age, years (SD)* Less than 9 years of education (%)	105 (51) 72.0 (12.2) 50 (24)	46 (41) 75.2 (11.2)
**Stroke subtype (%)**		
Cerebral infarction TIA Cerebral hemorrhage	164 (79) 28 (14) 16 (7)	
**Risk factors (%)**		
Hypertension* Hyperlipidemia Diabetes Cigarette smoking (present) Coronary heart disease Atrial fibrillation Daily alcohol use *BMI > 25*	123 (59) 117 (56) 23 (11) 18 (18) 45 (22) 65 (31) 20 (24) 119 (57)	
**TOAST classification (%)**		
Large-vessel disease Cardio-embolic disease Small-vessel disease Stroke of undetermined etiology	21 (10) 60 (29) 64 (31) 63 (30)	
**Assessments. Mean (SD) (n)**		
*IQCODE* *MMSE* *NIHSS* *BI* *mRS* TMT-A TMT-B 10-Word test immediate recall 10-Word test, delayed recall	3.1 (0.2) (n = 208)	
	26.0 (4.5) (*n* = 195) 2.44 (4.6) (*n* = 208) 17.5 (5.1) (*n* = 208) 1.5 (1.4) (*n* = 208) 73.7 (66.7) (*n* = 177) 152.8 (89.8) (*n* = 152) 17.9 (2.9) (*n* = 170) 4.3 (2.5) (*n* = 184)	25.8 (5.9) (*n* = 109) 1.02 (2.3) (109) 18.7 (3.7) (109) 1.4 (1.3) (109) 55.7 (39.6) (*n* = 101) 126.4 (67.7) (*n* = 82) 22.5 (8) (*n* = 106) 4.7 (3) (*n* = 105)

TIA = Transistent Ischemic Attack; Hyperlipidemia = total cholesterol > 5 mmol/L or LDL > 3 mmol/L; LDL = Low Density Lipoprotein; Diabetes = an established diagnosis or Haemoglobin A1C (HbA1c) ≥7.0; Coronary Heart Disease = previous myocardial infarction or present angina pectoris; BMI=Body mass index; TOAST = the trial of org 10,172 in acute stroke treatment classification; NIHSS=National Institute of Health Stroke Scale; BI=Barthel Activities of Daily Living Index; mRS = modified Rankin scale; IQCODE = the informant questionnaire on cognitive decline in the elderly. MMSE = Mini Mental State Examination; SD = standard deviation.* Hypertension = use of BP lowering drugs at baseline

**Table 2:** Characteristics at baseline, clinical cognitive profile and MRI findings at 1 year in stroke survivors by PET or no PET at 7 years

	PET	NO PET	***P*** value
N	26	85	
Male (%)	18 (69)	43 (51)	0.227
Mean age, years (SD)	72.9 (7.0)	75.9 (11.8)	0.239
Less than 9 years of education (%)	4 (15)	18 (21)	0.776
Living alone (%)	4 (15)	24 (28)	0.288
**Stroke subtype (%)**			0.926
Cerebral infarction	20 (77)	66 (78)	
TIA	4 (15)	11 (13)	
Cerebral hemorrhage	2 (8)	8 (9)	
**TOAST classification (%)**			0.724
Large-vessel disease	2 (8)	12 (14)	
Cardio-embolic disease	8 (31)	19 (22)	
Small-vessel disease	9 (35)	29 (34)	
Stroke of undetermined etiology	7 (27)	25 (30)	
**Assessments. Mean (SD) (n)**			
NIHSS	1.12 (2.1) (26)	1.99 (3.6) (85)	0.247
mRS	1.0 (1.2) (26)	1.26 (1.2) (85)	0.266
IQCODE	3.0 (0.1) (26)	3.1 (0.2) (85)	0.641
MMSE	27.8 (2.1) (26)	26.5 (4.0) (82)	0.113
**Clinical profile 12 months**			
Dementia	1 (26)	13 (84)	0.223
MCI	6 (26)	31 (84)	0.286
Normal cognition	19 (26)	40 (84)	0.040
**MRI 12 months. Mean (SD) (n)**			
MTLA grade	0.79 (0.8) (24)	1.18 (1.2) (77)	0.135
Fazekas score	1.52 (0.8) (25)	1.86 (0.9) (76)	0.095
Global Cortical Atrophy	1.28 (0.7) (25)	1.48 (0.6) (75)	0.177
CSF (Aβ)-42 (ng/L) 12 months (n)	840 (250) (13)	861 (302) (41)	0.824
CSF total tau (ng/L) 12 months (n)	270 (101) (13)	387 (192) (41)	0.040

TIA = Transient Ischemic Attack; TOAST = the trial of org 10,172 in acute stroke treatment classification; NIHSS=National Institute of Health Stroke Scale; BI=Barthel Activities of Daily Living Index; mRS = modified Rankin scale; IQCODE = the informant questionnaire on cognitive decline in the elderly; MMSE = Mini Mental State Examination; SD = standard deviation;MCI = mild cognitive impairment; MTLA = medial temporal lobe atrophy. CSF (Aβ)-42 = cerebrospinal fluid amyloid-β peptide

### Amyloid-binding in stroke survivors

The main characteristics, MRI, ^18^F-Flut-PET, and cognitive assessment findings are summarized in (see Additional file 1). Among the 26 patients who agreed to PET, five patients had no MRI at 7 years, required in our quantitative assessment method. Four scans were visually classified ^18^F-Flut(+). 13 patients were diagnosed with CI (mixed or neurodegenerative disease) at 7 years, one with a positive PET scan. Quantitative ^18^F-Flut-PET SUVr in different cortical regions is presented in Table 3.

**Table 3:** Quantitative ^18^F-Flutemetamol PET SUVr in different cortical regions

Cases	precuneus and posterior cingulate combined	anterior cingulate	prefrontal	inferior parietal	lateral temporal	Composite SUVr
1	–	–	–	–	–	
2	–	–	–	–	–	
3	0.82	0.70	0.60	0.64	0.55	0.65
4	–	–	–	–	–	
5	0.48	0.47	0.46	0.46	0.44	0.50
6	0.55	0.56	0.52	0.51	0.51	0.57
7	0.54	0.48	0.48	0.48	0.48	0.54
8	0.54	0.52	0.50	0.51	0.49	0.55
9	0.85	0.68	0.68	0.75	0.65	0.74
10	–	–	–	–	–	
11	0.52	0.51	0.49	0.49	0.48	0.54
12	1.24	1.21	1.21	1.14	1.16	1.13
13	0.54	0.49	0.50	0.51	0.49	0.55
14	–	–	–	–	–	
15	0.51	0.48	0.47	0.49	0.47	0.53
16	0.57	0.54	0.51	0.53	0.50	0.57
17	0.64	0.64	0.56	0.59	0.57	0.63
18	0.59	0.63	0.57	0.53	0.51	0.58
19	0.51	0.50	0.46	0.47	0.46	0.52
20	0.48	0.45	0.44	0.45	0.43	0.49
21	0.50	0.48	0.46	0.46	0.46	0.51
22	0.51	0.47	0.48	0.49	0.50	0.54
23	0.49	0.49	0.48	0.48	0.47	0.50
24	0.51	0.50	0.47	0.46	0.48	0.53
25	0.56	0.53	0.57	0.50	0.53	0.59
26	0.54	0.54	0.51	0.48	0.50	0.54

Comparing patients according to normal or CI at 7 years, no difference in mean SUVr (SD) was observed (0.57 (0.08) vs 0.60 (0.18), *p* = 0.54). Eight patients changed from normal cognition at 1 year to CI at 7 years, one of whom was amyloid positive. When comparing cognitive decline to stable cognition during follow-up, no difference in mean SUVr (SD) was observed (0.65 (0.24) vs 0.56 (0.07), *p* = 0.16). Three of four patients with visually positive PET had normal cognition at 7 years.

Eighty patients died before follow-up, 33 with CSF from 1 year. Logistic regression assessing age-adjusted CSF Aβ_42_ levels and death, did not demonstrate any significant association (OR 1.0, 95%CI 0.99–1.0). Age-adjusted CSF T-tau was not significantly associated with death (OR 1.0, 95%CI 1.0–1.0).

### Correlations

**Fig. 2:**
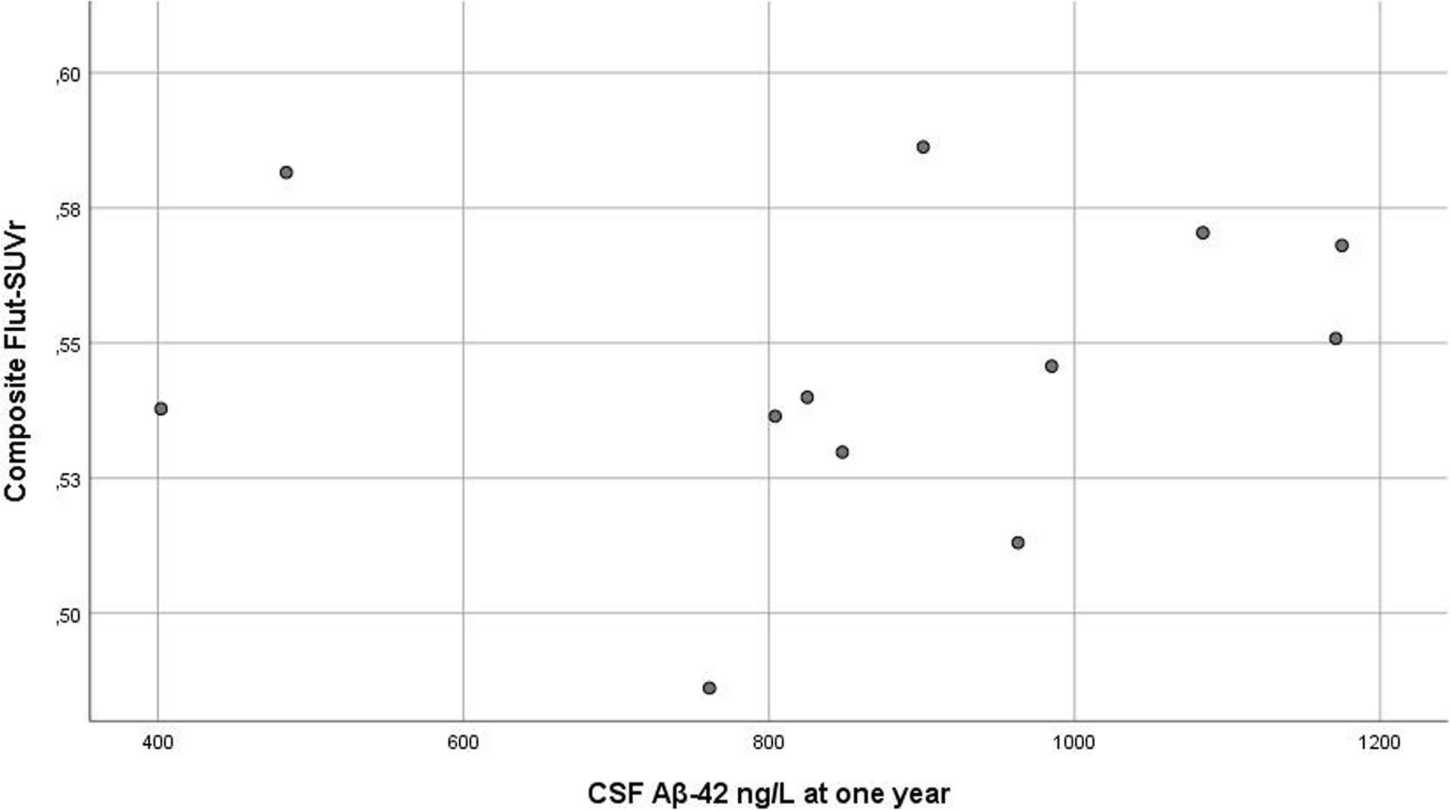
Associations between composite ^18^F-Flut-PET SUVr and CSF Aβ_42_ levels at 1 year

^18^F-Flut-PET SUVr at 7 years did not correlate with CSF Aβ_42_ levels at 1 year (r = 0.12; *p* = 0.703), MTLA (r = 0.10; *p* = 0.670), GCA (r = 0.10; *p* = 0.652) or MMSE (r = − 0.32; *p* = 0.162) at 7 years, when adjusted for age. TMT-A at 7 years was significantly correlated to ^18^F-Flut-PET SUVr (r = 0.69; *p* = 0.00), but exploring data revealed one outlier on the scatterplot, and after exclusion, there was no significant correlation. Associations between composite ^18^F-Flut-PET SUVr and CSF Aβ_42_ levels at 1 year are presented in Fig. 2.

## Discussion

In this cohort, only one patient diagnosed with CI 7 years post stroke was amyloid positive. Quantitatively assessed ^18^F-Flut-PET did not correlate with amyloid-β peptide (Aβ_42_) levels in CSF at 1 year or MTLA, GCA, MMSE, or TMT-A at 7 years.

Our findings with only one visually ^18^F-Flut(+) in post-stroke CI patients (8%) are in line with the results from the prospective DEDEMAS study (Determinants of Dementia After stroke), where only 2 out of 38 (5%) post-stroke MCI patients had positive amyloid scans. In the DEDEMAS study, neuropsychological testing was performed only 6 months after stroke, theoretically too early to capture amyloid deposition initiated by the stroke. As in our study, only a minor portion of patients completed the PET examination (38/178) [37].

Visual classification of ^18^F-Flut-PET is still the only validated method for clinical use, however quantification is beneficial to obtain a continuous measure of ^18^F-Flut-PET. One proposed cut-off for quantitatively assessed ^18^F-Flut-PET using CortexID, with pons as reference region, is SUVr 0.61 [65]. All patients with visually positive PET in our study had composite SUVr > 0.61 in line with the cut-off, even though we applied a different software for quantification. Amyloid deposition increases with age [12], and in our cohort the oldest had the highest SUVR and were the only visually PET positive among participants with CI.

Roberts et al. studied the prevalence of PET amyloid-positivity in a non-demented cohort drawn from the general population. They found that amyloid positivity in persons with MCI ranged from 0% in the age group 50–59 to 16% in participants aged 80–89 years [12]. Previous studies have reported a slightly higher prevalence between 10 and 30% in cognitively healthy-people [66, 67]. The frequency of amyloid positivity in our cohort is consistent with the observations from the general population, so no evident stroke-related inflammation with amyloid deposition is observed in our cases. Two cases had larger strokes with NIHSS ≥4 at discharge, both without amyloid positivity after 7 years.

When assessing PET semi- quantitatively, PET SUVr did not differ substantially between cases, and there was no significant difference when comparing those with or without CI, or when comparing those who experienced cognitive decline or stable cognition during follow-up. As our patients were imaged in regular clinical routine, with variable start in image acquisition, we acknowledge that this may affect the SUVr.

Three patients were diagnosed with MCI AD in consensus, none with amyloid positivity. This is in line with a previous study from the CAST cohort, where we found a correlation between pathological cerebrospinal fluid (CSF) concentrations of microtubule-associated protein tau (T-tau) 1 year post-stroke and brain atrophy, indicating that tau-linked neurodegeneration might be more important than amyloid post-stroke [68]. Recent studies suggest that changes in the blood-brain barrier (BBB) and BBB-mediated neurodegeneration, plays an essential role in cognitive dysfunction independent of Aβ biomarkers [69, 70].

In the study by Roberts et al., more amyloid-positive participants died compared to amyloid negative during the 4 year follow-up period, 13% vs 4.6% respectively [12]. Death before follow-up might bias our findings, but when assessing the relation between CSF biomarkers at 1 year and prospective mortality using logistic regression analysis, CSF Aβ_42_ at 1 year was not associated with death in our cohort.

As shown in Fig. 2, surprisingly, CSF Aβ_42_ at 1 year did not correlate with PET SUVr at 7 years. Three patients had pathological levels of CSF Aβ_42_ at 1 year, all without amyloid positivity at 7 years. Contrary to CSF T-tau, CSF Aβ_42_ levels remain unchanged after stroke when followed for 6 months [71]. One possible explanation could be that stroke decreases the ability to eliminate amyloid, maybe due to changes in the BBB [72], with normalization after some years. Garcia-Alloza et al. [73] demonstrated Aβ formation in a mice model as a transient phenomenon induced by a stroke, most likely through interference with amyloid clearance pathways. One study with repeated amyloid PET in 21 patients, one and 18 month post-stroke, found a significant reduction in amyloid accumulation in the infarct region, probably caused by a breakdown of BBB in the acute phase, with a leak of the radioactive ligand with “false” high SUVr [32]. The optimal timing for Aβ assessment post-stroke is not known but is essential for the development of future prediction models and individualized management. Due to small numbers, our observations must be confirmed in larger samples and interpreted with caution.

Our study has several limitations. First our limited sample size, as only 56 patients agreed to lumbar puncture at 1 year and 26 to PET after 7 years. Patients on anticoagulation therapy or those unable to consent, were excluded from lumbar puncture due to ethical considerations. This might bias our findings. After 7 years, due to time consuming examinations and long travel distances between hospitals, only highly motivated patients, and more of those with normal cognition at 1 year, agreed to PET, making our findings less generalizable. The strength of our study is the long-term follow-up with the same team of nurses and physicians, and measurement of amyloid at two time points. As far as we know, this is the first study to offer PET examination 7 years post-stroke, and by that adding important knowledge to amyloid evaluation post-stroke.

## Conclusions

In conclusion, amyloid binding was not common in our cohort of stroke survivors diagnosed with CI. Assessed quantitatively, amyloid load correlates neither with other measures related to neurodegeneration nor with CSF Aβ_42_ at 1 year in this cohort. Therefore, amyloid pathology may not be a key mediator of neurodegeneration 7 years post-stroke. Validations of our findings in larger studies are needed.

## Supplementary information

**Additional file 1 : Table 3.** Clinical characteristics, imaging features and CSF Aβ-42 of patients with PET examination

## Data Availability

The datasets used during the current study are available from the corresponding author on reasonable request**.**

## Abbreviations

AD: Alzheimer’s disease
BMI: Body mass index
CAST: Cognition After STroke
CI: Cognitive impairment
COWA: Controlled Oral Word Association Test
CSF Aβ_42_: Amyloid-β peptide-42
CSF: Cerebrospinal fluid
CT: Computer tomography
ECG: ECG
GCA: Global cortical atrophy
IQCODE: Informant Questionnaire on Cognitive Decline in the elderly
MCI: Mild cognitive impairment
MMSE: Mini Mental State Examination
MoCA: Montreal Cognitive Assessment
MRI: Magnetic resonance imaging
mRS: Modified Rankin Scale
MTLA: Medial temporal lobe atrophy
NIHSS: National Institutes of Health Stroke Scale
OCSP: Oxfordshire Community Stroke Project classification
p-ADL: Barthel Activities of Daily Living Index
PET: Positron emission tomography
P-tau: Phosphorylated microtubule-associated protein tau
RCT: Randomized controlled trial
SUVr: Standardized uptake value ratios
TIA: Transient ischemic attack
TOAST: The Trial of Org 10172 in Acute Stroke Treatment
TMT A: Trail Making Test A
TMT B: Trail Making Test B
T-tau: Microtubule-associated protein tau
VaD: Vascular Dementia
^18^F-Flut: ^18^F-Flutemetamol
