# Supplementary material for: No evidence for amyloid pathology as a key mediator of neurodegeneration post-stroke - a seven-year follow-up study
Source: BMC Neurol. 2020 May 8;20:174. doi: 10.1186/s12883-020-01753-w (PMC7206753; doi:10.1186/s12883-020-01753-w)
Supplement: Supplementary file 1 — Additional file 1 : Table 3. Clinical characteristics, imaging features and CSF Aβ-42 of patients with PET examination [file 12883_2020_1753_MOESM1_ESM.docx]

**Table 3.** Clinical characteristics, imaging features and CSF Aβ-42 of patients with PET examination

| **Cases** | **Age range/**  **Gender**  Baseline | **Stroke types/**  **Infract location/**  **NIHSS discharge** | **Cognitive evaluation**  Year 1 | Year 7 | **MTA**  Year 7 | **GCA**  Year 7 | **MSSE**  Year 7 | **TMT-A**  Year 7 | **Amyloid**  Visual  assessment  ^18^F-Flut(+) | **Binding Year 7**  Semi- quantitative  assessment  Composite SUVr, max | **CSF**  (Aβ)-42  (ng/L)  Year 1 |
| --- | --- | --- | --- | --- | --- | --- | --- | --- | --- | --- | --- |
| 1  2  3  4  5  6  7  8  9  10  11  12  13  14  15  16  17  18  19  20  21  22  23  24  25  26 | 66-70 f  66-70 m  66-70 m  71-75 m  71-75 f  61-65 m  56-60 m  61-65 m  66-70 m  71-75 m  66-70 m  76-80 f  71-75 m  71-75 m  56-60 m  46-50 m  61-65 m  66-70 f  61-65 f  61-65 f  71-75 m  61-65 m  51-55 f  61-65 m  56-60 m  66-70 f | Cortical, left side, NIHSS 4  Lacunar, left side, NIHISS 0  Lacunar, posterior, NIHSS 0  Lacunar, right side, NHISS 0  TIA, right side, NHISS 0  Cortical, right side, NIHSS 10  Cortical, right side, NHISS 2  Lacunar, posterior, NIHSS 0  Cortical, left side, NIHSS 0  Undetermined, left side, NIHSS 2  TIA, left side, NIHSS 0  Lacunar, posterior, NIHSS 1  TIA, right side, NIHSS 1  Cortical, left side, NIHSS 0  Subcortical H, left side, NIHSS 1  TIA left side, NIHSS 0  Lacunar, posterior, NIHSS 0  Lacunar, left side, NIHSS 2  Cortical, left side, NIHSS 1  TIA, left side, NIHSS 0  Lacunar, right side, NIHSS 1  Lacunar, left side, NIHSS 0  Subcortical H, right side, NIHSS2  TIA, left side, NIHSS 0  Lacunar, right side, NIHSS 2  Cortical, left side, NIHHS 0 | Dementia VaD  Normal  Normal  Normal  MCI mix  MCI mix  Normal  Normal  Normal  Normal  Normal  Normal  Normal  Normal  Normal  Normal  Normal  MCI mix  Normal  Normal  MCI mix  Normal  Normal  MCI mix  Normal  MCI mix | Normal  MCI VaD  Normal  Normal  MCI mix  MCI VaD  MCI VaD  Normal  Normal  Normal  MCI mix  MCI mix  MCI VaD  MCI AD  MCI mix  Normal  MCI VaD  MCI mix  Normal  Normal  Normal  Normal  Normal  MCI AD  Normal  MCI AD | -  -  3.5  -  3.0  1.0  1.5  2.5  0.5  -  1.0  3.5  2.0  -  1.1  0.5  1.5  1.0  1.0  1.5  0.5  1.5  1.0  1.5  1.0  2.0 | -  -  2  -  2  1  1  1  1  -  2  2  1  -  1  1  2  1  1  1  2  1  1  1  1  1 | 27  29  28  30  27  26  27  29  30  29  29  27  28  28  27  28  26  27  29  30  29  30  29  28  28  27 | 68  71  44  37  72  61  32  30  41  48  35  93  29  37  31  24  50  43  28  39  30  31  26  30  33  45 | No  No  Yes  No  No  No  No  No  Yes  Yes  No  Yes  No  No  No  No  No  No  No  No  No  No  No  No  No  No | *  *  0.65, 0.82  *  0.50, 0.65  0.57, 0.76  0.54, 0.71  0.55, 0.69  0.74, 0.85  *  0.54, 0,71  1.13, 1.24  0.55, 0.72  *  0.53, 0.71  0.57, 0.73  0.63, 0.79  0.58, 0.71  0.52, 0.70  0.49, 0.64  0.51, 0.69  0.54, 0.72  0.50, 0.58  0.53, 0.71  0.59, 0.77  0.54, 0.70 | 402  -  -  -  -  1084  402  985  -  -  804  -  1171  -  848  1175  -  484  -  761  963  825  -  -  901  - |

*MRI missing; m=male; f=female; lacunar infarcts; subcortical small (<15 mm) infarcts in the distal distribution of deep penetrating vessels; cortical infracts affects regions of the cerebral cortex; TIA=Transient Ischemic Attack; NIHSS=National Institute of Health Stroke Scale; H=hemorrhage; MCI=mild cognitive impairment; mix=mixed disease; VaD= vascular disease; AD= neurodegenerative disease; MTA= medial temporal atrophy; GCA=global cortical atrophy; MSSE= Mini Mental State Examination ;TMT-A= trail making test A; (Aβ)-42= cerebrospinal fluid (CSF) amyloid-β peptide.
